# *In memoriam*: Theodor Hiepe (1929–2022)—great German scholar of parasitology

**DOI:** 10.1007/s00436-023-07791-w

**Published:** 2023-02-13

**Authors:** Kai Matuschewski, Richard Lucius, Georg von Samson-Himmelstjerna

**Affiliations:** 1 Dept. of Molecular Parasitology, Institute of Biology, Humboldt Universität zu Berlin, 10115 Berlin, Germany; 2 Institute for Parasitology and Tropical Veterinary Medicine, Freie Universität Berlin, 14163 Berlin, Germany

**Keywords:** Zoonotic parasitic diseases, Parasite control, Small ruminants, Applied parasitology

## Abstract

Theodor Hiepe (1929-2022) was an outstanding researcher, a world-renowned scientist, a dedicated teacher and a great mentor. During his scientific career, which spanned over 60 years, he made major contributions to many different fields of parasitology. With the passing of Dr. h.c. mult. Theodor Hiepe in September 2022 the scientific community suffered a great loss.

On September, 2, 2022, the scientific community lost a great scholar, a highly regarded scientist, and a passionate mentor, Prof. Dr. Dr. h.c. mult. Theodor Hiepe. Theo, as his friends and close coworkers called him, devoted his entire life to understand and teach‚ the essence (das Wesen) of parasitism (Hiepe 1971). Everyone, who had the pleasure of meeting him, perceived him as a great thinker who has helped us understand more about the parasitic life style, the phenomenal complexity and inventory to adapt to animal hosts, the vast diversity of parasite species and life history traits, and the translational implications for parasite control.

Theodor Hiepe was born in Weimar, Germany in 1929. After forced military service as a teenager towards the end of World War II and imprisonment following the liberation, he moved to Leipzig, former German Democratic Republic, to study veterinary medicine, where he later worked as an assistant at the animal hospital. In 1956, he was hired as chief veterinarian at the Leipzig Zoological Garden, and at that time initiated his scientific work on the control of ectoparasites, helminths, and coccidia of farm and zoo animals, with an emphasis on small ruminants (Hiepe 1956; Hiepe et al. 1959). Four years later, at the young age of 31, Theodor Hiepe was appointed to the Chair of Parasitology and Veterinary Zoology of the Faculty of Veterinary Medicine at the capital university, Humboldt Universität zu Berlin. He succeeded Alfred Borchert, who was renown for his surveys on diseases of bees. Theodor Hiepe initiated a broad research agenda and established four working groups, namely protozoology, helminthology, arachno-entomology, and diagnostics.

Throughout his 35 years as head of the parasitology institute, Theodor Hiepe maintained a particular scientific interest in translating laboratory findings to parasite control and understanding immune processes in parasite infections. Theodor Hiepe’s main research areas were zoonotic parasitic diseases, parasite control strategies, and parasitic diseases of small ruminants, e.g., sheep and goats. His research was marked by his persistence, attention to detail and rigorous standards.

Early on, parasite diagnostics were a major mandate of the institute, and on average 1000 samples/month were processed and examined. In the 1960s, infections with *Dictyocaulus viviparus* (bovine lungworm) amounted to annual losses of thousands of young cattle, since well-tolerated anthelmintics or the highly effective, radiation-attenuated larval bovine lungworm vaccine (Jarret et al. 1958) were not yet readily available (Hiepe 1964). Similarly, appr. 75% of the livers of grazing cattle and sheep were infested with *Fasciola hepatica* (liver fluke) (Hiepe und Grünwoldt 1965). Theodor Hiepe’s institute developed integrated parasite control approaches combining hygiene measures, presumptive treatment with antiparasitic drugs and on-farm training (Hiepe 1961; Hiepe et al. 1963).

Likewise, long-lasting control of large flies of the genus *Hypoderma* (warble flies) became increasingly important largely due to their economic impact on cattle and small ruminants. After an impressive survey of over 150,000 heifers and cows to establish the seasonal dynamics of *Hypoderma bovis* infestation in cattle and treatment efficacy (Mieth et al. 1969), a control program based on a mathematical model was developed in cooperation with practicing veterinarians. In iterative steps, first in individual cattle herds, followed by municipalities, counties, districts, and eventually the entire country, eradication of the *H. bovis* was successful in as few as 4 years (Hiepe et al. 1974). This accomplishment prompted a large-scale bilateral project between the former German Democratic Republic and Mongolia, which was financed for 20 years with the aim to eradicate hypodermosis and market Mongolian rawhide globally (Hiepe et al. 1973; Hiepe and Splisteser 1976). Although the ambitious goal of country-wide eradication of major ectoparasites on-farm animals was not met, control of *H. bovis* and *Hypoderma lineatum* was very successful and established the foundation for national control programs (Ribbeck et al. 1979).

Theodor Hiepe took great pride in that neither he nor any of his coworkers participated in opportunistic political activities and stayed clear of any party memberships. He intuitively and positively followed Hermann Hesse’s belief that the antagonist of the intellectual is the party member, who can be put into service again and again. Strikingly, thanks to his international recognition and merits, he was permitted to travel regularly to congresses and meetings in Western Europe. His numerous academic and scientific merits include honorary doctorates from the University of Veterinary Medicine in Vienna, Austria and the University of Leipzig, Germany. He was a leading member of the German Academy of Natural Scientists Leopoldina, the actual National Academy of Sciences, and a founding member of the Berlin-Brandenburg Academy of Sciences after the fall of the Berlin Wall.

Two parasite species were named after him: *Eimeria hiepei*, a hepatic coccidian parasite in the American mink (*Neovison vison*) (Gräfner et al. 1967), and *Madathamugadia hiepei*, a filarial worm in Turner’s thick-toed gecko (*Chondrodactylus turneri*) in Southern Africa and likely transmitted by sand flies (*Phlebotominae*) (Hering-Hagenbeck et al. 2000).

Theodor Hiepe is widely recognized in the German-speaking community for his instructive academic textbooks. His first book on diseases of sheep (Hiepe 1970), which has continuously evolved over half a century (Bostedt et al. 2021), remains the authoritative textbook on diseases of small ruminants. Together with Regine Ribbeck, Renate Buchwalder, and Ruth Jungmann, he published a multi-volume textbook of parasitology (Hiepe et al*.* 1981; Hiepe et al. 1985; Hiepe and Ribbeck 1982; Hiepe and Jungmann 1983). Similarly, remembered is the more recent course book on general parasitology (Hiepe et al. 2006).

His scholarial and scientific achievements were furthermore acknowledged by the German Society for Parasitology who’s honorary member he became in 2012 and which awarded him with the prestigious Rudolphi and Leuckart medals in 1997 and 2000, respectively. In 1997, he also became an honorary member of the World Association for the Advancement of Veterinary Parasitology (WAAVP). Already in 1989 and in the still divided Berlin (and until to date the only time in Germany), he had organized with great success the biannual meeting of this global association of veterinary parasitologists (Eckert 2013).

He was an immensely intelligent man and an outstanding scholar. His passion for parasitology and his insights into parasite/host dynamics inspired generations of early career scientists. He will be sadly missed (Fig. 1).

**Fig. 1:**
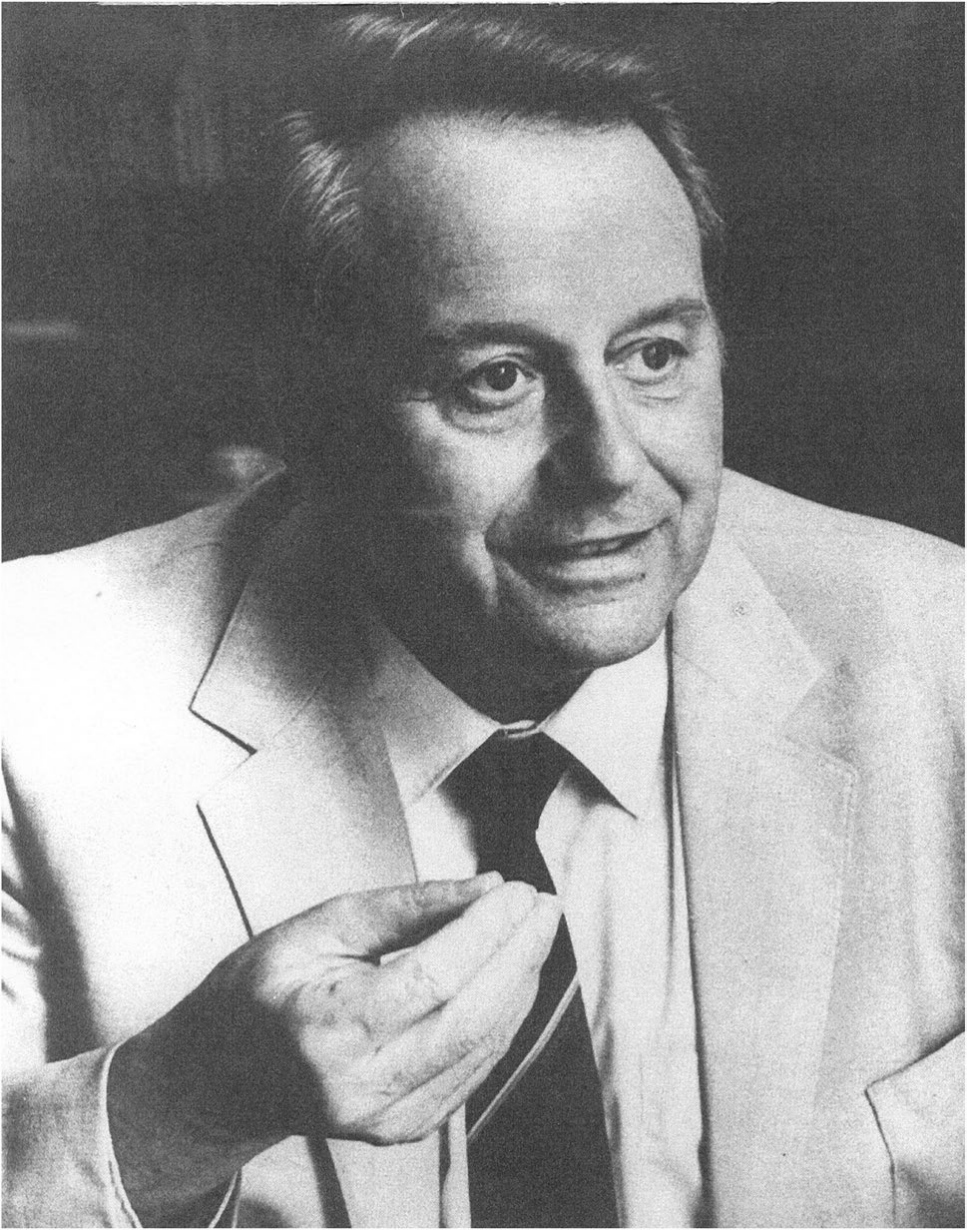
Theodor Hiepe (1929–2022)

## Data Availability

Access to the photograph of Prof. Dr. Dr. h.c. mult. Theodor Hiepe is available upon request.
